# Uterocutaneous fistula after cesarean section; Case report

**DOI:** 10.1016/j.ijscr.2021.106621

**Published:** 2021-11-19

**Authors:** Jinan Nori Hasan, Dildar Haji Musa, Ayad Ahmad Mohammed

**Affiliations:** a Duhok polytechnic University, Kurdistan Region, Iraq; b Department of Surgery, College of Medicine, University of Duhok, Kurdistan Region, Iraq

**Keywords:** Uterocutaneous fistula, Cesarean section, Fistula, Uterus

## Abstract

**Introduction and importance:**

Fistula is an abnormal tract communicating two epithelial surfaces, uterocutaneous fistula is an extremely rare. The classical presentation is cyclical bleeding from an abnormal opening in the scar of previous cesarean section.

**Case presentation:**

A 28 year-old was presented 6 months after cesarean section with history of severe pain and blood discharge during menstruation from the previous transverse supra-pubic scar. The patient had history of previous 2 cesarean sections. Abdominal examination revealed a localized tenderness at site of previous operation scar with 1*1 cm opening at the central part of the scar which was discharging blood during pressure over the lower abdomen. There was no urine discharge from the scar. Vaginal examination by the speculum was normal.

**Clinical discussion:**

Endometriosis was suspected and the patient received medical treatment with little improvement. Later, surgical intervention was done, the scar was excised with an abnormal fistulous tract connecting endometrial cavity at the site of the previous scar was found. Complete excision of the tract was done and the uterus was re-sutured using a slowly-absorbable suture material. The patient had no complications with regular cycles.

**Conclusion:**

Uterocutaneous fistula is rare and usually follow cesarean section, suturing the uterus with non-absorbable suture material is reported in this case to be one of the underlying causes, other causes include infection, necrosis, foreign bodies, or malignancy. The fistula tract must be defined and any associated infection controlled, complete resection of the fistulous tract and suturing the uterus with absorbable suture material is required.

## Highlights

• Causes of uterocutaneous fistulas are numerous like iatrogenic, endometriosis, malignancy, etc.
• The classical presentation is cyclical bleeding from the scar of previous cesarean section.
• A combined medical and surgical approach may be required.

## 1 Introduction

Fistula is defined as an abnormal tract that communicate between two epithelial surfaces; uterocutaneous fistula is an extremely rare clinical entity and occurs due to the presence of an abnormal tract that communicate between the uterine cavity and the skin. The exact incidence is not well estimated due to the rarity of this condition [1].

Causes of uterocutaneous fistulas are numerous including iatrogenic injuries during surgery, endometriosis, intrauterine devices, chronic infection, malignancies with local invasion to the adjacent organs, prolonged use of abdominal drains, radiation injury, trauma, and the incomplete closure of wounds particularly the uterine wall [2], [3].

The classical presentation is that patients present with cyclical bleeding from and abnormal opening in the scar of previous cesarean section [1].

Imaging modalities are helpful in the diagnosis which include magnetic resonance imaging (MRI) of the pelvis, hysterosalpingography, fistulography, hysteroscopy, computed tomography scan (CT scan) with intravenous contrast and the use of sagittal reconstruction is another helpful diagnostic tools. Ultrasonography have a limited role in the diagnosis [2].

The management depends on the underlying cause and usually directed to individual patients as there is no standard management plan. A combined medical and surgical approach may be required. Hormonal therapy in the form of GnRH (gonadotropin-releasing hormone) agonist will induce atrophic changes in the lining epithelium and may help to reduce the fistula discharge and the subsequent closure of the tract which is helpful in small size fistulas, however this is usually ineffective in large size of fistulas and when the skin opening is large. Surgical intervention is very effective and prompt rapid recovery, laparoscopic surgery may be adopted in selected patients [1], [2], [4].

The work has been reported in line with the SCARE 2020 criteria [5].

## 2 Patient information

A 28 year-old lady who was had previous 3 children (Gravida 3, Para 3) presented 6 months after cesarean section with history of severe pain and blood discharge during menstruation from the previous transverse supra-pubic scar, in between the menstrual cycles she complained from pus discharge from the same site of the scar. The patient had no dysuria with normal color urine, no frequent urination, and the defecation was normal.

The patient had history of previous 2 cesarean sections, the past medical history was negative for any chronic illnesses, and the cycles were regular with no history of dysmenorrhea.

The cesarean section was done at the 37th week of gestation as an emergency surgery because she had previous 2 scars and developed severe uterine contractions, she delivered a healthy male baby weighing 3.6 Kg.

The drug history was negative and the family history was negative for any relevant genetic information or psychosocial abnormalities.

### 2.1 Clinical findings

On physical examination she had normal vital signs, the patient was afebrile, with no pallor, and the body mass index was 28.

Abdominal examination revealed a localized tenderness at site of previous cesarean section scar with a 1*1 cm opening at the central part of the scar which was discharging blood during pressure over the lower abdomen. There was no urine discharge from the scar. Vaginal examination by the speculum was normal.

### 2.2 Diagnostic assessment

The complete blood count was normal with normal hemoglobin and white blood cells count, the urinalysis was also normal. The patient was sent for abdominal ultrasound which showed no abnormalities.

### 2.3 Therapeutic intervention

From the clinical scenario we suspected endometriosis as the provisional diagnosis as many patients present after cesarean section complaining from cyclical bleeding at the site of the scar. The patient received medical treatment in the form of GnRH agonists for three months with little improvement and the symptoms recurred after that.

**Fig. 1:**
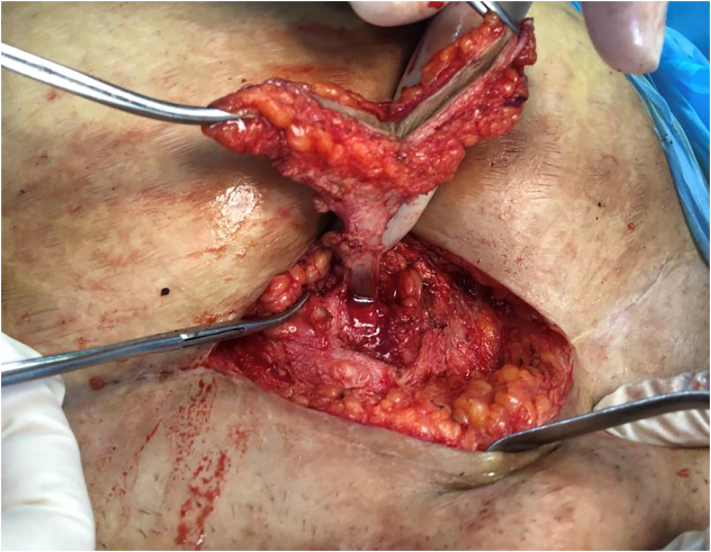
An intraoperative picture showing the fistulous tract between the skin and the uterus.

**Fig. 2:**
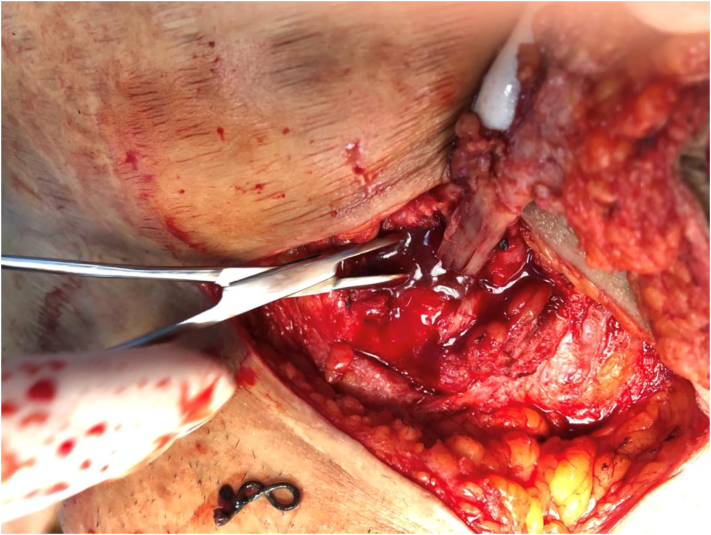
An intraoperative picture showing the fistulous tract between the skin and the uterus after removing the silk sutures.

The second option was surgical intervention, the operation was performed under general anesthesia, the scar was excised and there was an abnormal fistulous tract which was connected to the uterus and the endometrial cavity at the site of the previous uterine scar which was sutured by a non-absorbable suture material (Silk) at the time of previous surgery (Fig. 1, Fig. 2).

**Fig. 3:**
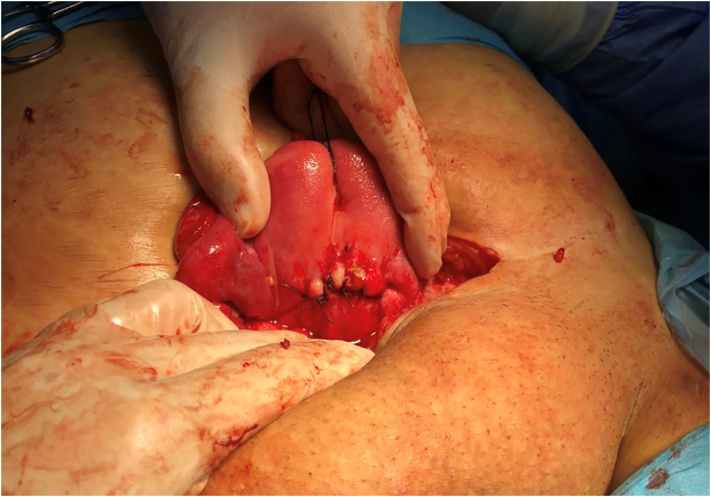
An intraoperative picture showing the site of the fistula in the anterior wall of the uterus after being sutured with an absorbable suture material.

Complete excision of the tract was done with removal of the previous sutures, removal of the necrotic tissue was done, refreshment of the edges was performed, the uterus was re-sutured using a slowly absorbable suture material, irrigation of the wound was performed with warm normal saline, and then the abdominal wall was closed (Fig. 3).

The operation was done by a gynecologist and a general surgeon.

### 2.4 Follow-up and outcomes

The immediate post-operative condition was smooth and satisfactory with no immediate complications. Follow up the patient was done for 8 months with no post-operative complications with normal regular menstrual cycles.

## 3 Discussion

Uterocutaneous fistula is rarely seen in clinical practice and usually occur after cesarean sections or other pelvic surgeries. Uterine fistulas occur more commonly with the urinary bladder and the bowel due to iatrogenic injuries, infections, and malignancies. There are some reported cases of uterocutaneous fistulas, the majority of them followed cesarean section [2], [6], [7].

Postoperative infection is a major implicating factor for the development of uterocutaneous fistula, sometimes necrosis of the anterior uterine wall may occur due to excessive suturing or infection. Most cases occur in the postpartum period or in the postoperative period, the time of presentation after surgery is not well defined and each patient have a variable presentation. Other causes include endometriosis which cause fistula without previous history of surgery or interventions [6], [8].

The diagnosis is usually clinical one when the patient present with cyclical bleeding with menses from the previous lower abdominal wound or chronic purulent discharge of pus or serosanguineous fluid. The injection of methylene blue through the cervix is very helpful when the diagnosis is not very clear, it shows the spillage of the dye from the wound in cases of patent fistulous tract. Imaging can be helpful in detecting the abnormal tract between the uterine cavity and the skin, magnetic resonance imaging (MRI) or CT scan with contrast agents are very helpful to define the anatomical planes in the pelvis. Fistulography or hysterosalpingography are also very informative which involve the injection of the water soluble contrast material through the skin opening or through the cervix and will demonstrate the abnormal connection between the skin and the uterine cavity. Hysteroscopy will visualize the abnormal tract directly [2], [4], [9].

Our case presented 6 months after cesarean section due to the use of non-absorbable suture material (Silk) for suturing the uterus, so we can add the use of non-absorbable suture material for closure of the uterus as one of the causes behind the development of uterocutaneous fistula. The use of non-absorbable suture materials increase the risk of chronic infection and may lead to the development of fistula [10].

The management of uterocutaneous fistula is a stepwise and involve careful assessment of the patients general condition, investigations to detect the underlying cause, the anatomy of the tract must be identified clearly before any kind of intervention, associated infection must be treated with appropriate antibiotics and drainage of any abscesses, the best surgical management involve excision of the whole fistulous tract with suturing of the uterine wall with an absorbable suture material. Follow up is recommended for early detection of any postoperative event and early intervention when required [11].

## 4 Conclusion

Uterocutaneous fistula is rare and usually follow cesarean section, suturing the uterus with non-absorbable suture material is reported in this case to be one of the underlying causes, other causes include infection, necrosis, foreign bodies, or malignancy. The fistula tract must be defined and any associated infection controlled, complete resection of the fistulous tract and suturing the uterus with absorbable suture material is required.

## Sources of funding

The authors are the main source of funding.

## Informed consent

Written informed consent was obtained from the patient for publication of this case report and accompanying images. A copy of the written consent is available for review by the Editor-in-Chief of this journal on request.

## Patient's perspective

I was worried about the cause of this cyclical bleeding especially after failed medical treatment.

## Ethical approval

Not applicable.

## Research registration

Not applicable.

## Provenance and peer review

Not commissioned, externally peer-reviewed.

## Guarantor

Dr. Ayad Ahmad Mohammed is the guarantor of the work.

## CRediT authorship contribution statement

The concept of reporting the case and data recording is done by Dr. Jinan Nori and Dr. Dildar Haji. Drafting the work is done by Dr. Ayad Ahmad Mohammed. Final approval of the work to be published was done by Dr. Ayad Ahmad Mohammed.

## Declaration of competing interest

There is no any conflict of interest.
